# A review of diffuse hemispheric glioma, H3 G34-mutant disease development, DNA repair, microenvironment, and treatments on the horizon

**DOI:** 10.1038/s41698-025-01070-w

**Published:** 2025-08-14

**Authors:** Cameron Crowell, Ziwen Zhu, Yingxiang Li, Maria L. Varela, Quinn Ostrom, Patrick J. Cimino, Sohil H. Patel, Suzanne J. Baker, Chetan Bettegowda, Houtan Noushmehr, Claudia L. Kleinman, Laura Canty, Gustavo Alencastro Veiga Cruzeiro, Anandani Nellan, Oren Becher, Tom B. Davidson, Dolores Hambardzumyan, Adam Green, Robert Thorne, Kelli Wilson, Brett Theeler, Jason Gregory, Peter Mathen, Nadia Biassou, Sheila McThenia, Craig Erker, Robert Galvin, Ariane Soldatos, Pedro R. Lowenstein, Maria G. Castro, Sadhana Jackson

**Affiliations:** 1 Division of Hematology and Oncology, IWK Health Centre, Halifax, NS Canada; 2 Faculty of Medicine, Dalhousie University, Halifax, NS Canada; 3 Department of Neurosurgery, University of Michigan Medical School, Ann Arbor, MI USA; 4 Department of Cell and Developmental Biology, University of Michigan Medical School, Ann Arbor, MI USA; 5 Rogel Cancer Center, University of Michigan Medical School, Ann Arbor, MI USA; 6 Neurosurgery, Population Health Sciences, Duke University School of Medicine, Durham, NC USA; 7 Surgical Neurology Branch, National Institute of Neurological Disorders and Stroke, Bethesda, MD USA; 8 Radiology and Medical Imaging Department, University of Virginia Health, Charlottesville, VA USA; 9 Center for Excellence in Neuro-Oncology Sciences, Department of Developmental Neurobiology, St. Jude Children’s Research Hospital, Memphis, TN USA; 10 Department of Neurosurgery, Johns Hopkins University School of Medicine, Baltimore, MD USA; 11 Hermelin Brain Tumor Center, Department of Neurosurgery, Henry Ford Hospital, Detroit, MI USA; 12 McGill Centre for Translational Research in Cancer, Lady Davis Institute for Medical Research, McGill University, Montreal, QC Canada; 13 The Hospital for Sick Children, Toronto, ON Canada; 14 Department of Pediatric Oncology, Dana-Farber Boston Children’s Cancer and Blood Disorders Center, Boston, MA USA; 15 Pediatric Oncology Branch, National Cancer Institute, Bethesda, MD USA; 16 Pediatric Hematology, Mount Sinai Medical, New York, NY USA; 17 Cancer and Blood Disease Institute, Children’s Hospital Los Angeles, Keck School of Medicine, University of Southern California, Los Angeles, CA USA; 18 Oncological Sciences and Neurosurgery, Icahn School of Medicine at Mount Sinai, New York, NY USA; 19 Morgan Adams Foundation Pediatric Brain Tumor Research Program, Department of Pediatrics, University of Colorado School of Medicine, Aurora, CO USA; 20 Denali Therapeutics, South San Francisco, CA USA; 21 Division of Preclinical Innovation, National Center for Advancing Translational Sciences, NIH, Rockville, MD USA; 22 Uniformed Services University, School of Medicine, Bethesda, MD USA; 23 Radiation Oncology Branch, National Cancer Institute, Bethesda, MD USA; 24 NIH Radiation Department, Bethesda, MD USA; 25 Children’s Healthcare of Atlanta, Atlanta, GA USA; 26 Department of Pediatrics, Dalhousie University, Halifax, NS Canada; 27 Department of Pediatrics, Division of Pediatric Hematology-Oncology, University of Minnesota, Minneapolis, MN USA; 28 NINDS Consult Service, National Institutes of Health, Bethesda, MD USA

**Keywords:** CNS cancer, Cancer microenvironment

## Abstract

Diffuse hemispheric glioma H3 G34-mutant is primarily diagnosed in adolescents/young adults. While molecular diagnostics have improved, cellular mechanisms that drive tumor progression and therapy resistance are poorly understood. Combining previous published studies with findings from the 2024 NIH G34-mutant symposium aid in summarizing translational and clinical updates on disease development, cellular repair processes, and the immune microenvironment. This collective work is meant to outline opportunities for treatment and prolonged survival.

## Introduction

Diffuse hemispheric glioma (DHG), H3 G34-mutant is a highly aggressive and infiltrative brain tumor which predominately affects the adolescent and young adult (AYA) population. A pivotal advancement in the understanding of pediatric gliomas occurred over a decade ago when two studies identified mutations in the H3.3 histone^1,2^. These mutations, altering amino acid 34, primarily glycine-to-arginine (G34R) and less frequently glycine-to-valine (G34V), help define a new distinct subset of hemispheric gliomas in the latest World Health Organization (WHO) classification of central nervous system tumors^3^. While this new recognition aids in the diagnostic definition of DHG, H3 G34-mutant, its true prevalence is poorly defined.

Patients with DHG initially present after a short interval of symptoms suggestive of increased intracranial pressure, new onset seizures, and/or neurological/cognitive abnormalities. Diagnostically, these tumors are often found supratentorially via brain MRIs with imaging consistent with an infiltrative mass with variable contrast enhancement^4^. Confirmation of diagnosis is established after surgical intervention with molecular classification. Historically, treatment of all high-grade gliomas follow standard treatment for glioblastoma, IDH wild type; which consists of surgical resection, chemoradiotherapy, and subsequent chemotherapy for maintenance^5^. While DHG, H3 G34-mutant is associated with a poor median overall survival of 17.3–21.5 months, no targeted treatment regimen currently exists^6,7^. This underscores the urgent need for deeper insights into potential therapeutic targets.

This manuscript aims to provide a concise overview of our current understanding of DHG, H3 G34-mutant, including its disease development, DNA repair, microenvironment, as well as potential future treatment options. We will highlight the complexities of this glioma variant, drawing on the expertise and insights of the leading researchers in the field. Notably, this paper synthesizes discussions and insights from the National Institutes of Health (NIH) symposium titled “What about G34R/V?: Challenges and Opportunities.” This symposium brought together world-leading experts to review and discuss ongoing research on DHG, H3 G34-mutant within the basic, translational, and clinical fields.

## Disease development of DHG H3 G34-mutant

### Discovery of the G34 histone mutation

In 2012, Jabado et al. made a groundbreaking discovery concerning the molecular make up of pediatric high-grade gliomas^1,8,9^. They employed exome sequencing on 48 pediatric histologically-defined glioblastoma (GBM) samples, revealing somatic mutations in the H3.3 ATRX-DAXX chromatin remodeling pathway. Their research found that 44% of pediatric GBM tumors harbored somatic mutations in the H3.3 ATRX-DAXX chromatin remodeling pathway, with 31% of these tumors exhibiting recurrent H3-3A (previously named H3F3A) histone mutations resulting in H3.3 K27M or H3.3 G34R/V. Of this 31%, the majority harbor the K27M mutation, and a minority exhibited the H3 G34 mutation. These histone mutations were highly prevalent in children and young adults, signifying a distinct molecular profile in gliomas that correlated with alternative lengthening of telomeres. This association also suggested that chromatin architecture defects, due to histone mutations, play a pivotal role in the pathogenesis of pediatric and young adult GBM. Furthermore, ATRX-DAXX mutations were found to be nearly universally present in cases with H3 G34 mutations, adding to their molecular complexity^10^. Interestingly, the tumorigenic H3 G34 mutation has rarely been reported to demonstrate co-occurrence with the H3K27M onco-histone mutation, which classically presents with midline tumor location^11^. These findings suggest that the H3 G34 mutation represents a distinct molecular subtype of gliomas, with its own unique pathogenesis and clinical implications. Yet, the infiltrative nature of the G34-mutant has demonstrated ease in brainstem involvement (contiguous and non-contiguous spread) despite multi-modality treatments^12^.

That same year, Baker et al. highlighted the significance of histone H3 mutations in the development of pediatric gliomas^2^. While research done by Jabado had a broader focus, Baker et al. performed whole genome sequencing on seven brainstem diffuse midline gliomas, previously termed diffuse intrinsic pontine glioma (DIPG), and identified somatic H3.3 K27M mutations in H3-3A and H3.1 K27M mutations in H3C2 (previously named HIST1H3B). To validate these findings in a larger cohort and extend beyond DIPG, they performed targeted Sanger sequencing on the 14 genes encoding histone H3 isoforms in 43 DIPG and 36 non-brainstem pediatric high-grade gliomas. Not only did they discover that 22% of non-brainstem pediatric gliomas harbored mutations in H3-3A gene, or in the related H3C2 gene, but they also showed that up to 14% had somatic mutations causing an H3.3 G34R alteration. Furthermore, this mutation was found in none of the 50 brainstem gliomas that were analyzed and, again as described by Jabado et al., was completely exclusive from any H3 K27M alterations. Prior to these publications, no studies had identified recurrent somatic histone mutations in cancer.

These early findings provided key insights into the importance of histone mutations in high-grade gliomas, particularly pediatric-type gliomas, and contributed to the 2021 CNS WHO classification system^3^.

### Epigenetic profile

The H3 G34-mutations in DHGs are not merely spectators. These muations actively sculpt the tumor’s epigenetic landscape, imparting profound changes that influence tumor behavior and treatment response. A critical mechanism of action of the G34 mutations is their interference with standard histone modification patterns, which are crucial for normal cellular differentiation processes.

**Fig. 1:**
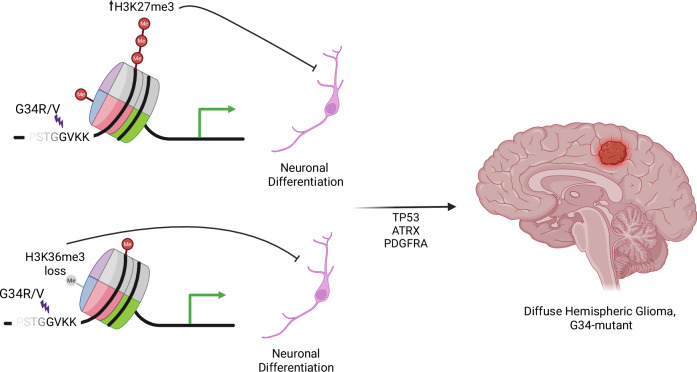
Epigenetic dysregulation in H3 G34-mutant DHG. Reduction or loss of H3K36me3 due to H3F3A G34-mutations can lead to an increase H3K27me3 levels, resulting in epigenetic silencing and influences on cell lineage development.

Specifically, the G34-mutations act in *cis* conformation, disrupting the normal deposition of H3K36me3 and H3K27me3 marks, primarily through inhibiting the SETD2 methyltransferase^13^. The blockade of SETD2 function impairs trimethylation of histone H3 on lysine 36, a key epigenetic marker essential for gene expression, transcription elongation, alternative splicing, and DNA repair^14,15^. Moreover, the reduction in H3K36me3 can indirectly affect the polycomb repressor complex 2 (PRC2) activity, which acts to define cell lineage development (Fig. 1). Typically, high levels of H3K36me3 inhibit the methylation of H3K27 to H3K27me3 by PRC2, thereby maintaining a balance between active and repressive chromatin states^16,17^. However, when H3K36me3 levels are reduced due to H3F3A G34-mutations, this leads to an inhibition of SETD2 methylation of H3K36, causing the disinhibition of PRC2, to increase H3K27me3 levels; a hallmark of epigenetic silencing. K27, on the other hand, has shown opposing dysregulation of the same set of genes with a reduction in PRC2 rather than an increase, suggesting that K27M mutations drive cells towards a less differentiated stem-like epigenetic state and the H3 G34-mutation drives cells towards a specialized neuronal state^18,19^.

This disruption is further complicated by interactions with histone lysine demethylase 4 (KDM4), leading to abnormal patterns of H3K36me3 distribution in G34-mutant tumors^15^. H3 G34 mutations can also deplete histone modifications in cis due to impaired DNM3TA (DNA methyltransferase 3α) recruitment^20^. O6-methylguanine-DNA methyltransferase (MGMT), which is downregulated in G34 mutant DHG, paradoxically increases tumor vulnerability to alkylating agents like temozolomide (TMZ), a standard chemotherapeutic drug^21^.

Recent studies identified multiple components of the Nucleosome Remodeling and Deacetylase (NuRD) complex as selective dependencies in H3 G34-mutant DHGs—dependencies not observed in other cancer types. Additionally, H3 G34-mutations disrupt phosphorylation at serine 31 (S31) on the H3.3 variant, an exclusive post-translational modification within the cell cycle. This disruption may further exacerbate genomic instability, posing additional challenges in the tumor’s maintenance of genomic integrity^22^.

The understanding of these epigenetic changes uncovers potential avenues for therapeutic intervention. Targeting the unique epigenetic landscape of DHG, H3 G34-mutant, through the development of drugs that can modulate specific histone modifications, could offer new routes in combating these resistant tumors.

### Molecular co-alterations

Beyond its characteristic histone mutations, our understanding of molecular co-alterations that arise in this tumor has grown rapidly. The H3 G34 mutation does not occur in isolation, but instead, G34-mutant gliomas frequently exhibit other well-known oncogenic changes. Nearly all cases demonstrate loss of OLIG2 expression, *ATRX* loss, and *TP53* mutation, resulting in unifying features of this tumor type^1,23^. Furthermore, these tumors also frequently display *MGMT* promoter hypermethylation, with occasional upregulation of *MYCN*, mutation of *NOTCH2NL*, and more seldom *EGFR* amplification^23–26^. In addition, mutations in MUC family genes, including *MUC16* and *MUC17*, have been shown to occur in about half of cases^27^. Despite these advances in our molecular understanding of this tumor, the prognostic implications of these mutational profiles remain up for debate. A recent study has also looked at tumor suppressor genes *PTEN* and *CDKN2A/B* (cyclin-dependent kinase inhibitor 2A/B), finding alterations in these genes to be more common in DHG, G34-mutant when compared to H3 K27M-altered gliomas^28^. It appears as though *TP53* mutations, *ATRX* loss, and *PDGFRA* alterations are fundamental to G34-mutant tumor development.

### Interneuron lineage hierarchy

Perhaps the most striking co-alteration that co-occurs with this tumor type is that of *PDGFRA*^27,29^. Recent advances in genomic and molecular research have elucidated the fascinating hierarchy and cellular origin of these tumors; highlighting their progression along an interneuron lineage^19,29^. In ~60–80% of DHG, H3 G34-mutant tumors, researchers have identified activating mutations in *PDGFRA* to be a key driver of tumorigenesis^29^. These mutations are particularly compelling as they tend to be recurrently selected in tumor relapses, underscoring their role in tumor survival and adaptability. Notably, the *PDGFRA* mutations do not act in isolation; rather, they interact with regulatory elements normally active in GSX2/DLX- expressing interneuron progenitors^29^.

Genetic-screened homeobox 2 (GSX2) and distal-less homeobox 1 (DLX1) are two transcription factors that influence the development of progenitor cells into ventral interneurons. Chromatin configuration in DHG, H3 G34-mutant expresses PDGFRA and the interneuron transcription factor of GSX2 adjacent to each other. Specifically, the open chromatin loop conformation favors the use of GSX2 regulatory components for the co-option of PDGFRA-dependent tumor growth^29^. Furthermore, in contrast to what is seen in DHG, H3 G34-mutant, in excitatory neurons, as well as in embryonic stem cells, the relationship between PDGFRA and GSX2 is more distant. This interaction facilitates the ectopic expression of PDGFRA, a phenomenon not observed in glial progenitors from the oligodendrocyte lineage, which are traditionally implicated in other forms of glioma. In addition to exhibiting a GSX2 and DLX1/2 positive signature, these tumors lack expression of transcription factors known to both excitatory neurons and those needed for oligodendrocyte development. As previously mentioned, OLIG2, a marker of oligodendrocyte lineage, is demonstrated to have absent immunoreactivity in these tumors, further aligning these tumors with an interneuron progenitor lineage. Ultimately, these features offer DHG, H3 G34-mutant, a unique epigenetic and transcriptional profile.

This insight aligns with observations that the tumor cells in DHG, H3 G34-mutant cells, spatially and transcriptionally resemble cellular structures referred to as ganglionic eminence, which are involved in GABAergic interneuron production during human brain formation. The understanding that these tumors originate from interneuron progenitors, instead of the more commonly assumed glial lineage, showcasing a crucial shift in the perception of glioma biology^19,29^. The G34R mutation is more prevalent than the G34V mutation in this disease context, suggesting specific nuances in tumor development and progression. Advancing this research avenue are large-scale genome-wide CRISPR screens that have identified several genes expressed in interneuron progenitors as critical dependencies for DHG, H3 G34-mutant tumor cell survival.

As such, these genes emerge as potential therapeutic targets. Among these, CDK6 has been spotlighted as a top gene dependency, selectively crucial for H3 G34-mutant gliomagenesis compared to other cancer types. Preclinical studies indicate that inhibition of CDK6, through targeted drugs or novel degraders, can push tumor cells towards a more mature, GABAergic interneuron-like state^19^. Other preclinical studies, targeting H3 G34-mutations in vivo to the ganglionic eminences, followed by in vitro drug screens, also uncovered specific sensitivity of these tumors to the FGFR inhibitor Infigratinib^30^. This suggests a strong therapeutic potential by reducing tumor proliferation and improving patient prognosis.

Overall, this hierarchical shift in DHG, H3 G34-mutant upon CDK4/6 inhibition suggests that combinatorial treatments that also target the GABAergic interneuron progenitor lineage (referred to as eIN-like cells) would provide new therapeutic avenues. However, it remains unclear to what extent eIN-like cells resemble terminally differentiated neurons and what role they play in the tumor microenvironment. Future research focused on disrupting specific lineage connections and exploiting the tumor’s lineage-specific vulnerabilities could lead to novel and more effective interventions.

## DNA repair pathways

### Genomic instability, DNA repair, and cGAS/STING activation

A hallmark of DHG, H3 G34-mutants is the accumulation of genomic instability, stemming from impaired DNA repair pathways. This defect is attributed to the mutation’s impact on histone modifications, which alter chromatin structure and reduce accessibility for DNA repair machinery^21^. As a result, DNA damage accumulates, leading to persistent genomic instability that fuels tumor progression.

One critical downstream consequence of this genomic instability is the activation of the cyclic GMP-AMP synthase (cGAS) and stimulator of interferon genes (STING) pathway. cGAS recognizes cytosolic DNA fragments, often originating from genomic instability or micronuclei, and activates STING, triggering a robust immune response. This pathway promotes the production of type I interferons and pro-inflammatory cytokines, which recruit and activate immune cells to the tumor site. The activation of the cGAS/STING pathway in G34R-mutant gliomas has been demonstrated in both mouse and human models, where elevated STING signaling correlates with enhanced innate immune responses^21^.

In the context of therapy, the cGAS/STING pathway offers a valuable target for amplifying anti-tumor immunity. Radiation therapy, combined with DNA damage response inhibitors, such as PARP inhibitors or CHK1/2 inhibitors, has been shown to increase DNA damage, further activating the cGAS/STING pathway and enhancing immune responses. Preclinical models reveal that these combination therapies can lead to significant tumor regression, long-term survival, and the establishment of immunological memory^29^. Importantly, the accumulation of micronuclei, while primarily serve as a biomarker of genomic instability, has been shown to contribute to the activation of cGAS/STING, highlighting the therapeutic potential of leveraging this mechanism^31^.

While *TP53* is a well-known tumor suppressor gene that is commonly mutated in various cancers, it specifically causes genomic instability by allowing cells with damaged DNA to continue dividing without proper repair. The addition of H3 G34-mutant histone further results in chromatin remodeling with replication stress and damaged DNA repair^21,32^. Giacomini et al. demonstrated that H3.3 mutant cells specifically prevent healing of S phase damage, resulting in non-homologous end joining (NHEJ) and improper repair^33^. They additionally found that G34R mutations exhibit treatment responsivity to camptothecins and PARP (poly (ADP-ribose) polymerase) inhibitors. Hawkins et al. also described the increased dependency of G34R mutant glioma cells on base excision repair (BER) proteins. They found that interrupting the BER process induces therapeutic repair vulnerability. Specifically, knockdown of BER pathway proteins or inhibition via the chemosensitizing agent, salinomycin, resulted in increased cell death and oxidative stress^33^.

**Fig. 2:**
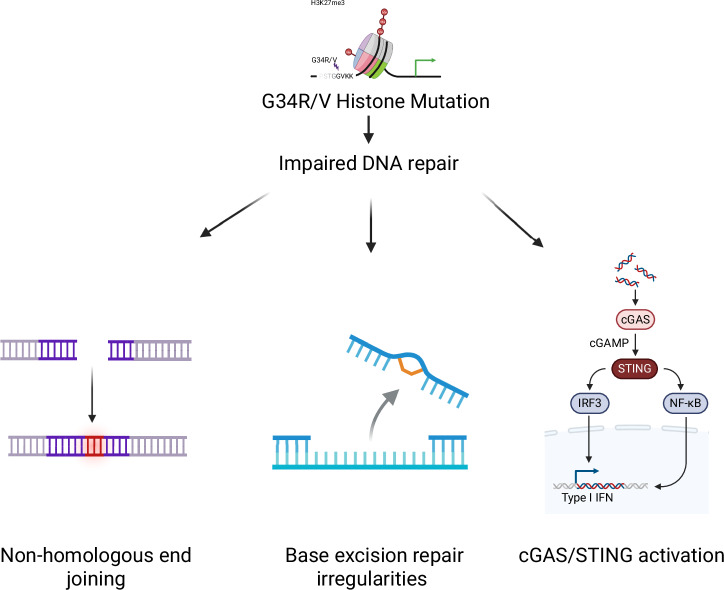
Genomic instability of H3 G34-mutant cells. G34-mutation results in impaired DNA repair with downstream consequences of non-homologus end joining (NHEJ), reliance on base excision repair proteins and activation of the cyclic GMP-AMP synthase (cGAS) and stimulator of interferon genes (STING) pathway.

Collectively, these findings demonstrate that while H3 G34 mutations cause genomic instability, future clinical studies of targeted agents that exploit these DNA damage repair vulnerabilities have the potential to enhance disease treatment while also inducing a robust microenvironmental immune response (Fig. 2). While targeted therapies exhibit greater specificity than standard oncologic treatments, several studies have demonstrated the high incidence of off-target kinase effects from PARP and CHK1/2 inhibitors^34,35^. Specifically, at micromolar concentrations varied PARP inhibitors can have differential off-targets to multiple kinases (e.g., PIM1, CAMK, CMGC, CDK9, AGC, etc.), and CHK1/2 inhibitors can also alter cyclin E activity; which makes planning for synergistic therapies with these agents quite challenging^35,36^. Additionally, use of these agents for prolonged periods can result in treatment resistance and resultantly tumor growth.

## Tumor immune characteristics

### An immune responsive landscape

The immune microenvironment in H3 G34-mutant gliomas differs significantly from that of other gliomas, with unique features that make these tumors more amenable to immune-based therapies. Single-cell RNA sequencing of G34R mutant gliomas reveals upregulation of pathways associated with interferon-gamma (IFN-γ) signaling, JAK/STAT activation, and immune cell recruitment^36^. Akin to several high-grade gliomas, these tumors exhibit reduced immunosuppressive components, including a marked decline in monocytic myeloid-derived suppressor cells (M-MDSCs) and M2-like tumor-associated macrophages. Simultaneously, there is an enrichment of activated microglia and increased infiltration of CD8+ cytotoxic T cells. Specifically, H3 G34R mutant cells secrete lower levels of cytokines such as G-CSF, VEGF, and TGFβ compared to H3.3 WT^37^. This alteration is linked to increased repressive histone marks (H3K27me3) at cytokine gene promoters in H3.3 G34R mutant cells. Additionally, conditioned media from H3.3 G34R mutant cells support CD8 T cell proliferation and higher IFNγ secretion, unlike media from H3.3 WT cells, which inhibit T cell proliferation^37^. Interestingly, CD11b+ Gr-1+ myeloid-derived suppressor cells (MDSCs) from H3 G34R tumors do not inhibit T cell proliferation, suggesting that this mutation reduces immunosuppressive mechanisms within the TME. In support of these findings, G34R-mutant gliomas also display an increased expression of major histocompatibility complex (MHC) class I molecules and upregulation of interferon-responsive genes^21^. These collective observations suggest that this histone mutation induces an “immune-permissive” state, potentially increasing the efficacy of immune-stimulatory therapies. Yet, for patients with DHG G34-mutant, there exists both interpatient and intrapatient heterogeneity of immune responses which are linked to tumor location, therapy exposure (e.g., steroid dosing, radiation dosing/timing, etc.), and degree of immune cell infiltration. The distinct immune landscape of G34-mutant tumors represents both a diagnostic hallmark and a therapeutic opportunity to target these tumors with immunotherapies. An additional unexploited therapeutic modality is the use of chimeric antigen receptor (CAR) cellular therapy against G34-mutant gliomas. A first-in-human trial of GD2-targeted CAR T-cell therapy achieved partial responses in diffuse midline glioma patients with H3K27 alteration, which demonstrated the feasibility of locoregional delivery of cellular therapy products to the central nervous system^38^. Cellular therapies are susceptible to tumor-induced exhaustion, but given the “immune-permissive” state of the G34-mutant tumor microenvironment, cellular therapy may also offer a promising treatment avenue; yet these studies are yet to be explored.

### Immune-mediated gene therapy: a paradigm shift in treatment

The development of immune-stimulatory gene therapy marks a promising shift in the treatment of H3 G34-mutant DHGs. These tumors have historically exhibited limited response to conventional treatments such as chemotherapy and radiation. However, recent advances have identified their unique immune-permissive tumor microenvironment (TME), making them an attractive target for immunotherapies. Among these, the thymidine kinase (TK)/Flt3 ligand (Flt3L) gene therapy has emerged as a powerful strategy to enhance anti-tumor immunity and prolong survival in preclinical models^37^. TK/Flt3L gene therapy capitalizes on this immune- permissive state by not only directly inducing tumor cell death but also by amplifying innate and adaptive anti-tumor immune responses through dendritic cell activation and cytotoxic T cell expansion^37^.

The therapeutic approach employs an adenoviral vector system to deliver two key immune-stimulatory genes: herpes simplex virus thymidine kinase (HSV-TK) and Flt3 ligand (Flt3L). HSV-TK serves as a suicide gene that converts the prodrug ganciclovir (GCV) into a toxic nucleotide analog, selectively killing tumor cells that express the gene. Importantly, this process generates a bystander effect, where neighboring tumor cells that do not express TK are also eliminated due to the diffusion of toxic metabolites through gap junctions^39^. The immune activation component of the therapy is driven by Flt3L, which acts as a potent dendritic cell (DC) recruiter and activator. DCs are critical for antigen presentation and play a pivotal role in orchestrating tumor-specific immune responses. Upon recruitment to the tumor site, they process dying tumor cells, present tumor-associated antigens, and activate cytotoxic CD8+ T cells, which then launch a targeted attack against remaining tumor cells^40^.

One of the reasons why TK/Flt3L therapy is particularly effective in H3 G34R gliomas is the increased expression of MHC class I molecules in these tumors, which facilitates more efficient antigen presentation and immune recognition. Additionally, transcriptomic analyses has demonstrated higher basal activation of interferon-gamma (IFN-γ) and JAK/STAT signaling

pathways, both of which are crucial for the amplification of immune responses^21^. This suggests that, unlike other DHGs that actively suppress immune activity, G34R-mutant tumors are primed for immune engagement. Once TK/Flt3L therapy is introduced, the tumor microenvironment rapidly shifts towards an immunologically active state, with increased recruitment of effector T cells and reduced suppressive myeloid populations.

**Fig. 3:**
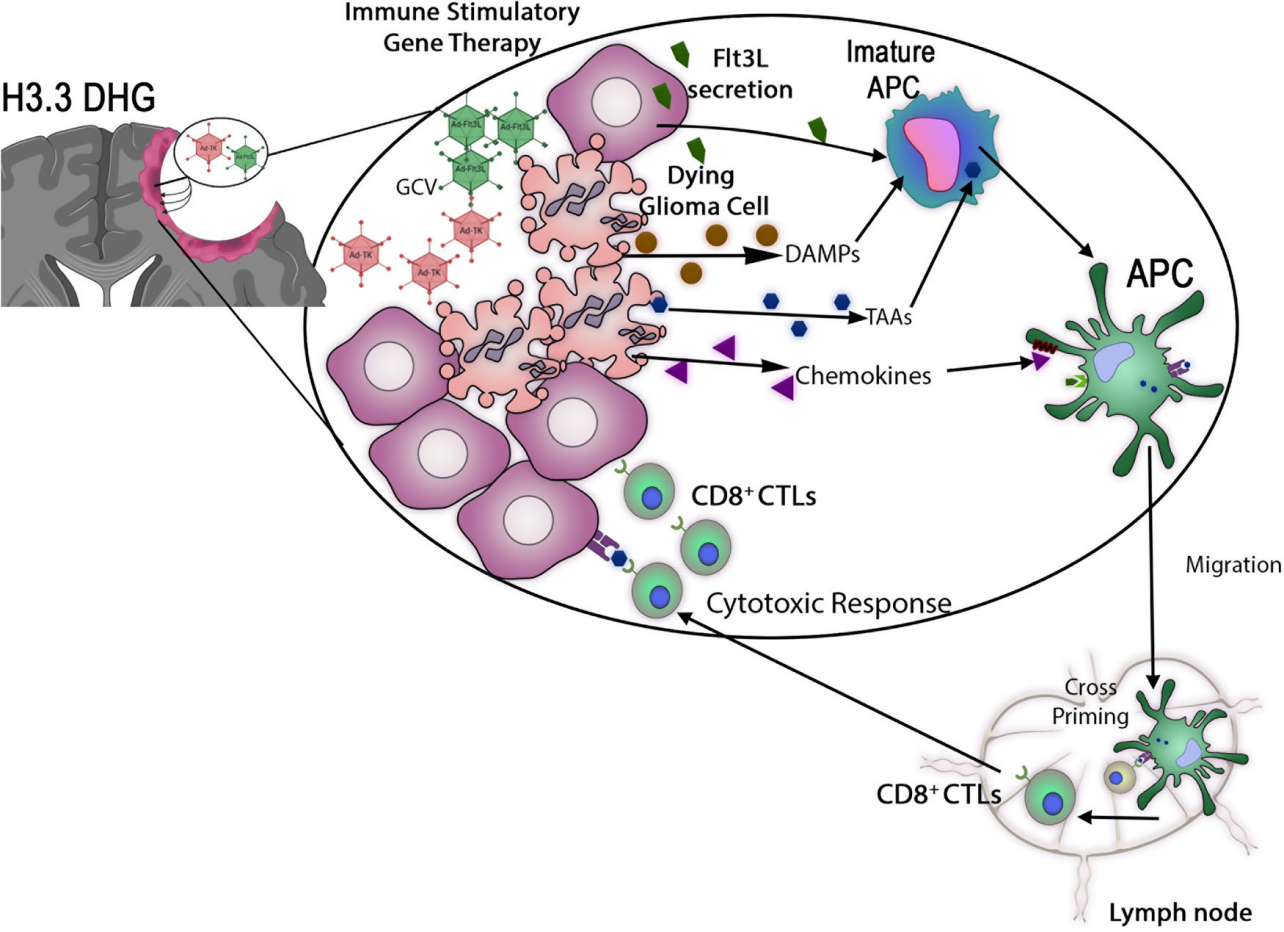
Detailed mechanism of immune-stimulatory gene therapy in diffuse hemispheric glioma (DHG). After surgical resection, adenoviral vectors encoding HSV1-thymidine kinase (Ad-TK) and FMS-like tyrosine kinase 3 ligand (Ad-Flt3L) are injected into the tumor cavity, followed by administration of the prodrug ganciclovir (GCV). Cells infected with Ad-Flt3L secrete Flt3L, which promotes the recruitment and differentiation of immature antigen-presenting cells (APCs). Tumor cells infected with Ad-TK phosphorylate GCV, which is further phosphorylated by cellular kinases to its tri-phosphorylated form, a purine analog. When this cell enters division, DNA synthesis is halted, leading to immunogenic cell death. This process results in the release of glioma-specific antigens, damage-associated molecular patterns (DAMPs), cytokines, and tumor-associated antigens (TAAs). APCs recruited into the brain by Flt3L will uptake the TAAs released from dying glioma cells, along with DAMPs and cytokines, which further stimulate immune responses. APCs loaded with glioma-specific antigens migrate to the lymph nodes, where they present the antigens to naïve T cells on MHC I molecules, resulting in cross-priming and clonal expansion of glioma antigen-specific effector T cells. These cytotoxic T cells infiltrate the tumor and kill glioma cells through a cytotoxic response.

In preclinical models, over 60% of treated mice exhibited long-term survival, and the majority of these survivors rejected tumor rechallenges, indicating the development of durable immunological memory^37^. This suggests that TK/Flt3L therapy does not simply act as a short-term tumor-killing strategy but also trains the immune system to recognize and eliminate tumor cells over time, reducing the likelihood of recurrence. Beyond its effectiveness in preclinical glioma models, the success of TK/Flt3L therapy aligns with findings from other studies exploring immune-stimulatory approaches in brain tumors. The first in human dose finding combination of TK/Flt3L through a phase I trial in newly diagnosed glioblastoma has been successfully completed (NCT01811992). This trial was reviewed and approved by the institutional review board at the University of Michigan Medical School (HUM00057130), and an investigational new drug application was granted by the US Food and Drug Administration (BB-IND 14574). These studies identified the maximally safe tolerated dose of the dual therapy injected into the post-surgical space. While initial findings provide promising results in eradicating multifocal gliomas and enhancing long-term survival for newly diagnosed glioblastoma patients, future research is required to determine the efficacy of identifying other sites for viral vector injection (e.g., ventricular, lumbar spine, etc.), providing additional doses to prolong durable responses and use of synergistic agents to further enhance the immune cell activation^41^. Additionally, this raises the possibility that this approach could be extended to other glioma subtypes, particularly those with immune-responsive features similar to H3 G34R tumors (Fig. 3).

Taken together, TK/Flt3L gene therapy represents a transformative approach for treating H3 G34R mutant gliomas, leveraging both direct tumor cytotoxicity and long-lasting immune responses. The unique immunological profile of these tumors provides a strong rationale for clinical translation, and ongoing research continues to explore ways to enhance its efficacy in DHG patients. Diffuse hemispheric glioma, including those harboring the H3 G34R mutation, present a major therapeutic challenge due to their diffuse infiltrative nature, which complicates complete surgical resection^12^. Additionally, these tumors exhibit resistance to traditional chemotherapeutic regimens, partly due to an altered epigenetic landscape that impacts DNA repair pathways and cellular metabolism^42^. This has necessitated the exploration of alternative strategies such as immunotherapy, which aims to mobilize the host immune system to recognize and destroy tumor cells.

## Discussion

### Potential therapeutic targets

Despite considerable advances in our understanding of this disease, treatment of DHG, H3 G34-mutant gliomas continues to rely on conventional approaches without a recognized standard of care. Historically, both pediatric-type and adult-type high-grade gliomas have been treated with surgery, radiation, and TMZ per Stupp trial of maximally safe surgical resection, whole brain radiation of 60 Gy combined with TMZ, followed by maintenance therapy of oral TMZ; which provides a survival benefit compared with surgery and radiation alone^5,7,43,44^. Often patients with H3 G34-mutant glioma have *MGMT* promoter methylation which is associated with a better response to alkylating therapies, such as TMZ^7,9^. However, recent discoveries point to potential avenues for targeted therapy as opposed to conventional chemotherapy regimens (Table 1). While no unique therapies have been determined specifically for DHG, H3 G34-mutant disease progression has been linked to *PDGRFA* mutation or amplification in tumors with glioblastoma-like histology. Yet, neither in the neoadjuvant setting nor post radiation therapy has *PDGFR* inhibition been shown to be advantageous in prolonging median event-free survival historically for patients with high-grade glioma. These older studies also were not equipped with our current knowledge to molecularly stratify patients; with intent to evaluate for potential differences in response signals^23^. Additionally, *CCND2A* (cyclin D2, key regulator of cell growth and replication) amplifications are highly prevalent in G34-mutant tumor tissue, especially those with primitive neuronal/neuroectodermal-like histology; questioning the efficacy of long-term cyclin-dependent kinase (CDK) inhibitor usage. Only in a case study of a 10-year-old patient with multiply recurrent DHG, H3 G34-mutant, *ATRX* mutated, treated with ribociclib (FDA-approved CDK4/6 inhibitor) 350 mg/m^2^/day was prolongation in survival for 17 months on therapy. These findings demonstrate the potential for combination therapies with CDK inhibition for a more personalized treatment plan^19^. Ongoing studies like NCT05413304, which examines efficacy of abemaciclib permeability within DHG and DMG patients and NCT06342908 details effects of peptide-pulsed dendritic cell vaccine for specifically for patients with DHG G34-mutant, explore real-time neuropharmacokinetics and immune activation in DHG; which has the potential to inform future larger prospective studies^45,46^. Ultimately, clinical trials of high-grade glioma have largely failed to molecular stratify patients, as entities such as G34-mutant glioma are relatively new, while the sample size in high-grade glioma trials conducted to date often prohibits the study of rare subtypes. Diffuse hemispheric glioma, G34-mutant is distinct in its cell lineage, epigenetic program, molecular features, and tumor microenvironment. Therefore, dedicated clinical trials are urgently needed to discern whether promising treatments are effective on a molecularly stratified basis.

**Table 1:** Outlined vulnerabilities for therapeutic targeting for DHG, H3 G34-mutant

Major biological vulnerability	Potential therapeutic avenues
Low immunoreactive microenvironment	Dual Ad-tk/Ad-flt3L gene therapy to activate cGAS/STING pathway, PARP inhibitors, CHK1/2 inhibitors
CCND2A (cyclin D2) amplification, CDKN2A/B deletion	Cyclin Dependent Kinase 4/6 inhibitor
Damaged DNA repair	Camptothecins, PARP inhibitors, CHK1/2 inhibitors
Base excision repair	Knockdown of BER pathway proteins via Salinomycin
*MGMT* promoter methylation	Alkylating agents
*PDGRFA* mutation or amplification	PDGFR inhibitor
G34R mutation	FGFR inhibitor
Interneuron progenitor	Cyclin Dependent Kinase 4/6 inhibitor to induce neuronal maturation

### Missing elements for advancements

Advancing the field with regard to discovery and treatment will require filling several missing gaps. Creation of an annotated registry that catalogs worldwide incidence of DHG, H3 G34-mutant, including imaging, pathology, genomics, epigenomics, and treatment response data will allow for better ease between clinicians to establish a standard of care treatment plan^47^. The incorporation of next-generation sequencing and methylation profiling in routine diagnostic workups will expand the ability to identify and recruit individuals with DHG, H3 G34-mutant into both biological and therapeutic research studies. Efforts such as the Children’s Oncology Group’s molecular characterization initiative and the Childhood Brain Tumor Network’s biorepository and sequencing initiative will increase the availability of multi-omic data sources for this understudied entity. The expansion of the Children’s Oncology Group’s adolescent and young adult molecular characterization initiative to include older glioma patients will also enhance data acquisition for this tumor type that crosses both pediatric and adult neuro-oncology spaces. While notable missing elements, authors recognize logistical hurdles exist with regard to data standardization, funding, and clinical and research expertise/availability across varied research institutions.

To further studies in the translational space, established criteria for and development of improved preclinical models (including humanized rodent models), which can be shared among groups, would be paramount to move treatments to human clinical trials. Ultimately, expansion of patient-derived models and development of isogenic, syngeneic, and other preclinical models that recapitulate the developmental and molecular features of DHG, H3 G34-mutant will permit studies that aim to investigate its biology and apply biologically rationale treatments. More cohesive connections among labs researching DHG, H3 G34-mutant would accelerate expansive drug screening and establishment of patient-derived cell models/organoids, which could assist in a better understanding towards durable therapeutic targets.

## Conclusion

While the discovery of DHG, H3 G34-mutant has helped to harmonize understanding of histone mutant gliomas, there remain many unknowns about disease development, DNA repair mechanisms, and optimal therapies. Thus, advancements focused on establishing (1) clinical registries with tissue procurement, (2) reliable preclinical models, and (3) solid partnerships between pediatric and adult oncology clinical teams will encourage optimized treatment collaborations and better understanding the intricacies of this disease.

## Data Availability

Data is provided within the manuscript.
